# Relationship Between Clinics Offering Telemedicine and Population Density in Japan: An Ecological Study

**DOI:** 10.1089/tmr.2023.0054

**Published:** 2024-04-05

**Authors:** Takashi Kuwayama, Kazuhiko Kotani

**Affiliations:** Division of Community and Family Medicine, Center for Community Medicine, Jichi Medical University, Shimotsuke-City, Japan.

**Keywords:** depopulation, online medical care, physician distribution, rural medicine, remote medicine

## Abstract

**Background:**

The number of clinics offering telemedicine in Japan has been increasing. Regional characteristics such as population density and the number of physicians may be associated with the provision of telemedicine. This study investigated the relationship between clinics offering telemedicine and such regional characteristics for each prefecture in Japan.

**Methods:**

Data were collected from publicly available information that included the percentage of clinics offering telemedicine (real-time synchronous type) among all clinics (in 2022), population density, and the number of physicians for each of Japan's 47 prefectures. An ecological study was carried out to determine the correlation between the percentage of clinics offering telemedicine and regional characteristics for each prefecture, and Pearson correlation analysis and multiple regression analysis adjusted for regional characteristics were performed.

**Results:**

The min–max and mean levels were, respectively, 3.4–39.2% and 15.6% of clinics offering telemedicine, 66.6–6402.6 and 657.1 people per square kilometer of population density, and 185.2–356.7 and 274.0 physicians per 100,000 people. Geographically, the northeastern regions appeared to show a high percentage of clinics offering telemedicine relative to the southwestern regions. There was a significant negative correlation between the percentage of clinics offering telemedicine and population density (*r* = −0.31, *p* < 0.05; *β* = −0.31, *p* < 0.05).

**Discussion:**

The negative relationship of the provision of telemedicine in clinics with population density throughout Japan might be a reflection to ensure residents' access to clinics in less populated areas. Although further detailed studies are needed to confirm this, population density might be a useful measure for considering whether to offer telemedicine in clinics in Japan.

## Introduction

Telemedicine between physicians and patients through online devices is one of the options for diagnosis and treatment in the clinical setting.^1^ Telemedicine has become increasingly popular in recent years, yielding health benefits similar to those of face-to-face care.^2^ Several favorable effects on health care, including optimizing hospitalization and preventing the spread of infection, are reported in telemedicine.^3^ Telemedicine is also suggested to be cost effective.^4^

Telemedicine is becoming increasingly available in Japan, whereas the factors that contribute to the promotion of telemedicine should be further explored.^1^ In Japan, patients have free access to health care that is provided from clinics to hospitals.^5^ Most clinics are privately operated, and they typically provide primary care and outpatient care with either no beds or <20 beds.^5^ In general, telemedicine (real-time synchronous type) is offered by clinics in Japan, and clinics that offer telemedicine are required to notify the local government of telemedicine services.

A Japanese study has reported that clinics in urban areas became more interested in telemedicine during the coronavirus disease 2019 (COVID-19) pandemic.^1^ Considering the different distributions of physicians and clinics between rural and urban areas throughout Japan,^6^ telemedicine might be helpful in overcoming barriers to access, including distance and travel time, to clinics in rural areas.^7^ Thus, regional characteristics such as population density and the number of physicians may affect the consideration about whether to provide telemedicine.

To date, no studies have shown the provision of telemedicine in considering such regional characteristics. Therefore, in this study, we investigated the relationship between clinics offering telemedicine and regional characteristics for each prefecture in Japan.

## Methods

### Data

In Japan, consisting of 47 prefectures, the following publicly available data for each prefecture were collected: total population, population density, number of physicians per 100,000 people, number of clinics, number of clinics offering telemedicine, and percentage of clinics offering telemedicine. The total population and population density for each prefecture were obtained from the 2020 Population Census of the Statistics Bureau of Japan (Appendix A1). The number of physicians was obtained from the Statistics on Physicians, Dentists and Pharmacists for 2020 of the Ministry of Health, Labour and Welfare in the Japanese government (Appendix A2). The number of physicians per 100,000 people was calculated from the total population and the number of physicians in each prefecture.

The total number of clinics was obtained from the current survey of medical facilities of the Ministry of Health, Labour and Welfare in the Japanese government (Appendix A3). The number of clinics offering telemedicine was taken from the list of medical institutions that submitted notifications to local health and welfare bureaus in 2022 (Appendix A4). The percentage of clinics offering telemedicine was calculated by dividing the number of clinics offering telemedicine by the total number of clinics in each prefecture.

### Analysis

The distribution of clinics offering telemedicine by prefecture was plotted, and an ecological study was conducted to determine the correlation between the percentage of clinics offering telemedicine and regional characteristics (population density and the number of doctors per 100,000 people) for each prefecture, and Pearson correlation analysis and multiple regression analysis adjusted for regional characteristics were performed. For the distribution, geographic information including prefectural boundaries was collected from the Ministry of Land, Infrastructure, Transport and Tourism's Global Map of Japan Data (ver. 2.2) and mapped using QGIS, a software of Quantum Geographic Information System (ver. 3.22).

Pearson correlation analysis and linear multiple regression analysis were performed using EZR (Easy R),^8^ a graphical user interface for R (ver. 4.0.3; The R Foundation for Statistical Computing, Vienna, Austria). Owing to non-normality, population densities were log transformed and analyzed. The level of statistical significance was set at 5%.

All data used in this study were obtained from public sources and no personal information was included; therefore, the ethical review board waived the requirement for ethical approval in this study.

## Results

**FIG. 1.**
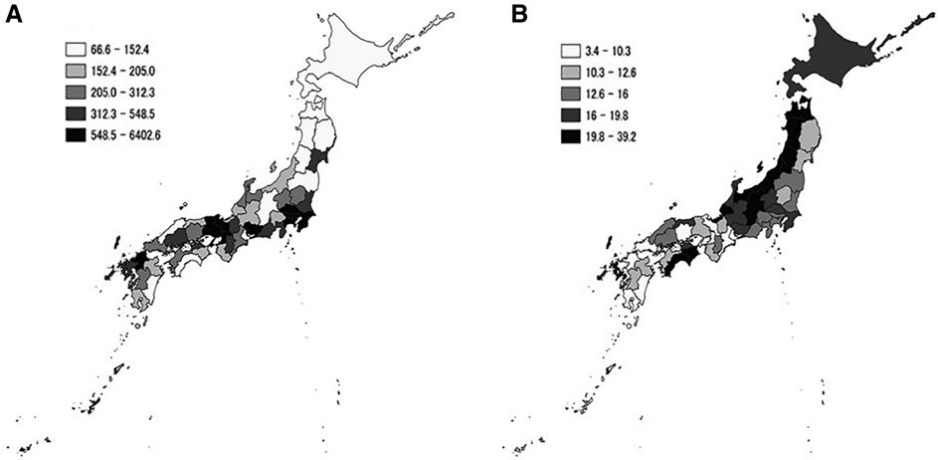
Geographical distribution of population density and percentage of clinics offering telemedicine. **(A)** Distribution of population density (people/km^2^). **(B)** Distribution of percentage of clinics offering telemedicine (%).

As given in Table 1, the min–max and mean levels for population density were 66.6–6402.6 and 657.1 people per square kilometer, respectively. The distribution appeared to be high in the central and southwestern areas relative to the northeastern areas (Fig. 1A). The min–max and mean number of physicians were, respectively, 185.2–356.7 and 274.0 per 100,000 people.

**Table 1.** National Distribution of Clinics Offering Telemedicine and Regional Characteristics

Prefectures	Number of clinics (***n***)	Number of clinics offering telemedicine (***n***)	Percentage of clinics offering telemedicine (%)	Population density (people/km^2^)	Number of physicians per 100,000 people (***n***)
All					
Minimum	484	44	3.4	66.6	185.2
Maximum	14,560	1869	39.2	6402.6	356.7
Mean	2232	314	15.6	657.1	274.0
Each prefecture					
Hokkaido	3432	583	17.0	66.6	262.8
Aomori	864	172	19.9	128.3	224.0
Iwate	886	111	12.5	79.2	223.0
Miyagi	1741	208	11.9	316.1	258.5
Akita	817	188	23.0	82.4	254.7
Yamagata	904	354	39.2	114.6	244.2
Fukushima	1390	176	12.7	133.0	215.9
Ibaraki	1782	269	15.1	470.2	203.6
Tochigi	1479	168	11.4	301.7	246.9
Gunma	1578	288	18.3	304.8	244.2
Saitama	4495	840	18.7	1934.0	185.2
Chiba	3868	626	16.2	1218.5	213.2
Tokyo	14,560	1869	12.8	6402.6	342.2
Kanagawa	7076	1120	15.8	3823.2	231.4
Niigata	1680	336	20.0	174.9	218.2
Toyama	759	183	24.1	243.6	273.7
Ishikawa	887	165	18.6	270.5	307.8
Fukui	578	169	29.2	183.0	270.5
Yamanashi	752	103	13.7	181.4	259.4
Nagano	1607	530	33.0	151.0	254.7
Gifu	1632	320	19.6	186.3	231.5
Shizuoka	2762	384	13.9	467.2	227.7
Aichi	5617	939	16.7	1458.0	236.6
Mie	1526	92	6.0	306.6	242.8
Shiga	1141	120	10.5	351.9	247.3
Kyoto	2492	84	3.4	559.0	355.1
Osaka	8763	703	8.0	4638.4	299.1
Hyogo	5198	630	12.1	650.5	276.9
Nara	1225	187	15.3	358.8	287.7
Wakayama	1023	125	12.2	195.3	318.8
Tottori	484	92	19.0	157.8	338.1
Shimane	711	95	13.4	100.1	314.1
Okayama	1615	111	6.9	265.4	333.1
Hiroshima	2545	385	15.1	330.2	278.8
Yamaguchi	1226	97	7.9	219.6	274.4
Tokushima	701	160	22.8	173.5	356.7
Kagawa	855	148	17.3	506.3	303.7
Ehime	1197	125	10.4	235.2	288.2
Kochi	530	161	30.4	97.3	333.3
Fukuoka	4805	440	9.2	1029.8	326.8
Saga	698	72	10.3	332.5	301.3
Nagasaki	1342	306	22.8	317.7	335.2
Kumamoto	1480	157	10.6	234.6	311.5
Oita	964	111	11.5	177.2	299.9
Miyazaki	916	92	10.0	138.3	269.2
Kagoshima	1384	117	8.5	172.9	293.0
Okinawa	921	44	4.8	642.9	264.9

The min–max and mean number of clinics offering telemedicine were, respectively, 44–1869 and 314, and the min–max and mean percentages of clinics offering telemedicine were 3.4–39.2% and 15.6%, respectively. Geographically, the northeastern areas appeared to show a high percentage of clinics offering telemedicine relative to the southwestern areas (Fig. 1B).

Pearson correlation analysis and linear multiple regression analyses between the percentage of clinics offering telemedicine and the number of physicians per 100,000 people showed no significant correlation, whereas there was a significant negative correlation between the percentage of clinics offering telemedicine and population density (*r* = −0.31, *p* < 0.05; *β* = −0.31, *p* < 0.05) (Table 2).

**Table 2.** Correlations for the Percentage of Clinics Offering Telemedicine

Variables	Univariable analysis		Multivariable analysis	
	***r***	***p***	***β***	***p***
Number of physicians per 100,000 people (*n*)	−0.13	0.37	−0.14	0.34
Population density (people/km^2^)^a^	−0.31	<0.05^b^	−0.31	<0.05^b^

*r*: Pearson correlation coefficient; *β*: standardized regression coefficient.

^a^ Lg-transformed variable.

^b^ Statistical significance.

## Discussion

In this study, the percentage of clinics offering telemedicine appeared to be high in the northeastern areas of Japan relative to the southwestern areas, indicating its regional disparities (broadly saying, high in east and low in west). Moreover, of note, a negative correlation was found between the percentage of clinics offering telemedicine and population density. For promoting telemedicine, it may be important to highlight population density, a unique regional characteristic.

Possible explanations for their relationship are considered. Telemedicine is reported to be useful in areas with long distance and travel time to clinics,^7^ and this may be the same in Japan as well. Usually, public transportation is limited in prefectures with low population density, where patients are sparsely distributed and each clinic must serve patients over a large area.^9^ Furthermore, looking around Japan, elderly people tend to live in areas with low population density.^10^ In prefectures with low population density, the elderly population may have greater difficulty accessing clinics than elderly people living in more urbanized areas.^10^

In such cases, telemedicine may be a suitable alternative to home medical care, in which physicians should long travel to patients' homes. Telemedicine could be a more efficient means of providing medical care in such situations, possibly leading to the results of this study.

The regional disparities in clinics offering telemedicine in Japan (as high in east and low in west) are also thought to be related to population density. Conversely, the population density is low in the northeastern areas of Japan and high in the southwestern areas (Table 1). For instance, the northeast areas, Hokkaido and Tohoku, have a mean value of 131.5 people per square kilometer, whereas the southwest areas, Chugoku, Shikoku, Kyushu, and Okinawa, show a mean value of 280.5 people per square kilometer. However, as multiple factors remained to be considered for the regional disparities, we may explore the additional explanations in future study.

It is assumed that Japan's population will continue to decline and consequently the number of areas with low population density will increase.^10^ Based on the findings of this study, population density might be a useful measure in predicting the spread of or recommending the provision of telemedicine. In other words, population density can serve as a measure for considering whether to provide telemedicine in clinics in Japan. Given that introducing telemedicine is often expensive,^11^ administrative and financial support for telemedicine may be more needed in prefectures with less populated density.

Although the findings of this study are reliable because publicly open and official data were used, we must take care of some limitations. First, an ecological study cannot fully confirm causality. Second, since there were no data regarding whether and to what degree clinics used telemedicine, the study might not completely reflect the actual states of use of telemedicine. Third, the effects of telemedicine on medical care and patient outcomes were not examined. Finally, whether or not the findings are seen in other countries (with different regional characteristics) than Japan must be investigated for generalizability. Future studies should take these details into account.

## Conclusion

This study revealed the negative relationship of the provision of telemedicine in clinics with population density throughout Japan. This might be a reflection to ensure residents' access to clinics in less populated areas. Although further detailed studies are needed to confirm this, population density might be a useful measure for considering whether to offer telemedicine in clinics in Japan.

## Abbreviations Used

COVID-19: coronavirus disease 2019

## Appendix

### Appendix A1

Population census, data source: Statistics Bureau of Japan. (2020) 2020 Population Census: Population, Households, Residences, Foreigners, Elderly Households, Single-Mother/Father-Father-Father Households, Parent-Child Living Together, etc. Available from: https://www.stat.go.jp/data/kokusei/2020/kekka.html [Last accessed: April 4, 2023] (in Japanese)

### Appendix A2

Statistics of Physicians, Dentists and Pharmacists, data source: Ministry of Health, Labour and Welfare. (2020) 2020 Statistics of Physicians, Dentists and Pharmacists. Available from: https://www.mhlw.go.jp/toukei/saikin/hw/kaigo/service21/index.html [Last accessed: July 8, 2023] (in Japanese)

### Appendix A3

Survey of Medical Institutions, data source: Ministry of Health, Labour and Welfare. (2022) 2022 Survey of Care Service Facilities and Establishments. Available from: https://www.mhlw.go.jp/toukei/saikin/hw/iryosd/m22/is2205.html [Last accessed: July 8, 2023] (in Japanese)

### Appendix A4

List of medical institutions that submit notifications to local health and welfare bureaus, data source: Hokkaido Health and Welfare Bureau. (2022) List of Medical Institutions Approved for Notification. Available from: https://kouseikyoku.mhlw.go.jp/hokkaido/gyomu/gyomu/hoken_kikan/todokede_juri_ichiran.html [Last accessed: June 30, 2022] (in Japanese)

Tohoku Health and Welfare Bureau. (2022) List of Medical Institutions Approved for Notification. Available from: https://kouseikyoku.mhlw.go.jp/tohoku/gyomu/gyomu/hoken_kikan/documents/201805koushin.html [Last accessed: June 30, 2022] (in Japanese)

Kanto Shinetsu Health and Welfare Bureau. (2022) List of Medical Institutions Approved for Notification. Available from: https://kouseikyoku.mhlw.go.jp/kantoshinetsu/chousa/kijyun.html [Last accessed: June 30, 2022] (in Japanese)

Tokai-Hokuriku Health and Welfare Bureau. (2022) List of Medical Institutions Approved for Notification. Available from: https://kouseikyoku.mhlw.go.jp/tokaihokuriku/newpage_00349.html [Last accessed: June 30, 2022] (in Japanese)

Kinki Health and Welfare Bureau. (2022) List of Medical Institutions Approved for Notification. Available from: https://kouseikyoku.mhlw.go.jp/kinki/gyomu/gyomu/hoken_kikan/shitei_jokyo_00004.html [Last accessed: June 30, 2022] (in Japanese)

Chugoku-Shikoku Health and Welfare Bureau. (2022) List of Medical Institutions Approved for Notification. Available from: https://kouseikyoku.mhlw.go.jp/chugokushikoku/chousaka/shisetsukijunjuri.html [Last accessed: June 30, 2022] (in Japanese)

Shikoku Health and Welfare Branch Bureau. (2022) List of Medical Institutions Approved for Notification. Available from: https://kouseikyoku.mhlw.go.jp/shikoku/gyomu/gyomu/hoken_kikan/shitei/index.html [Last accessed: June 30, 2022] (in Japanese)

Kyushu Health and Welfare Bureau. (2022) List of Medical Institutions Approved for Notification. Available from: https://kouseikyoku.mhlw.go.jp/kyushu/gyomu/gyomu/hoken_kikan/index_00007.html [Last accessed: June 30, 2022] (in Japanese)
